# Determinants of stroke among adult hypertensive patients on follow up in Addis Ababa public hospitals, Ethiopia: A case control study

**DOI:** 10.1371/journal.pone.0286845

**Published:** 2024-09-03

**Authors:** Feyisa Teshome, Bayisa Abdissa Baye, Wekashi Wili, Meseret Ifa, Guta Kune

**Affiliations:** 1 Moyale General Hospital, Oromia Regional State, Moyale Town, South Ethiopia; 2 Department of Public Health, College of Medicine and Health Sciences, Ambo University, Ambo, Ethiopia; 3 Department of Epidemiology, Faculty of Public Health, Jimma University, Jimma, Ethiopia; Main Line Health Care, UNITED STATES OF AMERICA

## Abstract

**Background:**

Stroke is a non-communicable disease that causes sudden global or focal neurological disorders. It is one of the major public causes of morbidity and mortality in low- and middle-income countries, including Ethiopia. Early identification of the determinants and prompt intervention remains critical to reduce morbidity and mortality from stroke.

**Objective:**

The study aimed to identify determinants of stroke among adult hypertensive patients on follow up in Addis Ababa public hospitals, Ethiopia.

**Methods:**

Unmatched case-control study design was conducted among 326(109 cases and 217 controls) study participants in Addis Ababa public hospitals from September 1–30, 2021. The cases were adult hypertensive patients who developed a stroke and the controls were adult hypertensive patients. The study participants were selected by consecutive sampling technique. Pretested structured interviewer assisted questionnaire and checklist were used to collect data. Data were entered into Epi data version 3.1; exported and analysed by SPSS version 23. All independent variables with p-value < 0.25 in the bi-variable logistic regression analysis were entered into multivariable logistic regression analysis. Finally, variables with p-value <0.05 were considered as determinants of the stroke.

**Results:**

In this study, current cigarette smoker(AOR = 5.55, 95% CI: 2.48, 12.43), current alcohol drinker(AOR = 4.27, 95% CI: 1.94, 9.38), medication non-compliance(AOR = 3.23, 95% CI: 1.62, 6.44), uncontrolled systolic blood pressure (AOR = 3.42, 95% CI: 1.64, 7.16), uncontrolled diastolic blood pressure(AOR = 4.29, 95% CI: 2.06, 8.93), high low density lipoprotein(AOR = 6.89, 95% CI: 3.57, 13.35) and diabetic mellitus(AOR = 3.25, 95% CI: 1.58, 6.69) were more likely to develop a stroke.

**Conclusion and recommendation:**

Cigarette smoking, alcohol use, non-adherence to medication, uncontrolled systolic pressure, uncontrolled diastolic blood pressure, high low-density lipoprotein, and diabetic mellitus were determinants of stroke. Providing health education about lifestyle changes and the consequences of hypertension at all follow-up is very important.

## Introduction

Stroke or cerebrovascular accident is clinically defined as a focal or global neurological disorder due to impaired cerebral perfusion or spontaneous haemorrhage in the brain, lasting 24 hours or more onset and resulting in death with no apparent cause other than vascular origin [1]. There are two pathologic types of stroke namely; ischemic stroke is the most common type and results from decreased blood supply to brain tissue due to blood clot formation and haemorrhagic stroke; which occurs due to rupture of blood vessels in the brain [2]. Stroke is the second leading cause of death worldwide after ischemic heart disease and the third leading cause of disability-adjusted life years (DALYs) after neonatal disease [3].

According to the Global Burden of Diseases, Injuries, and Risk Factors Study (GBD) report, there were 12.2 million stroke cases, 6.55 million stroke deaths, and 143 million stroke DALYs in 2019. Of this, 86% of deaths and 89% of stroke-related Disability Adjusted Life Years(DALYs) are in developing countries, which is about 3.8 times higher than in developed countries [4]. Recent data from the World Health Organization report shows that stroke accounted for 4.71% of all deaths in Ethiopia in 2014 and 39.57 (6.23%) of all deaths in 2017, with an age-adjusted mortality rate of 89.82 per 100,000 of the population, indicating an increasing burden of stroke in the country [5, 6].

The global incidence of stroke has increased by 25% among 20–64 year olds [7]. It is the leading cause of hospital admissions in Sub-Saharan Africa(SSA), accounting for approximately 15% of all hospital admissions and is a leading cause of death in both rural and urban settings [8]. Similarly, it is the leading cause of hospital admissions in Ethiopia(23.6%) of all neurological admissions and is emerging as a serious community health problem [9]. The 2012 surveillance of adult mortality in Addis Ababa found that stroke accounted for 11% of all deaths from NCDs [10].

Although the burden of stroke is worldwide, the damage is much greater in resource-limited continents like Africa. Due to the poor and under-developed healthcare system, rapid urbanization and ever-changing social and economic pressures leading to an increased prevalence of hypertension, diabetes, and obesity, all of which are major risk factors for stroke [11].

Stroke affects the most economically productive members of society, as well as the fact that young adults in low-income countries are more prone to stroke due to increased exposure to behavioural risk factors [12]. It also places a great financial and psychological burden on patients and their families [13]. Stroke is a growing clinical and economic burden costing approximately $103.5 billion annually in the United States [14] and without intervention, annual costs are projected to increase to $240.67 billion by 2030 [14]. Age, sex, race, uncontrolled blood pressure, diabetes, non-adherence to antihypertensive medication, high-fat diet, smoking, heavy alcohol consumption, low physical activity, obesity, and dyslipidaemia have previously been identified as determinants of stroke [15–22]. Although various modifiable risk factors are gaining importance, hypertension remains the leading cause of stroke globally and nationally [23]. And it is the most preventable neurological disease by controlling modifiable risk factors either through healthier lifestyle or medication [24].

Ethiopia has made significant progress in reducing adult morbidity and mortality by adopting a comprehensive national guideline for the clinical and programmatic management of no communicable diseases including stroke in 2016. In addition, WHO advises adults to get at least 150 up to 300 minutes of moderate-intensity aerobic physical activity for at least 75 up to 150 minutes of vigorous aerobic physical activity; or an equivalent combination of moderate and vigorous activity during the week [25].

Despite interventions, mortality from stroke remains one of the public health problems in the country [5]. Depending on the environment and methods used, the common determinants of stroke may differ from region to region, and particularly in country with diverse culture, socioeconomic characteristics and delays in reaching care health facility are challenging [10, 22, 26, 27].

Although previous studies identified determinants of stroke in some parts of Ethiopia, there is limited data in the study area that accounts for differences in socioeconomic status, access to treatment and it is useful for designing contextual interventions [9, 28]. Therefore, this study aimed to identify the determinants of stroke in adult hypertensive patients on follow-up in public hospitals in Addis Ababa.

## Methods

### Study design and setting

Hospital-based unmatched case-control study was conducted among hypertensive patients on follow up in public hospitals of Addis Ababa from September 1–30, 2021. Addis Ababa is the capital city of Ethiopia and currently, the total population projected from the 2007 census, is 5,006,000 with an annual growth rate of 4.42% in 2021. In the city, there were 96 health center 13 public hospitals, 28 private hospitals, and 882 clinics [29].

### Study participants

All adult (≥18 years of age) hypertensive patients who were on follow-up in Addis Ababa public hospitals during the data collection period were the source population. In this study, subjects were categorized into cases and controls. Adult hypertensive patients with stroke who were diagnosed by a neurologist (consultant internist) or confirmed by brain imaging (CT-scan or MRI) were defined as cases and adult hypertensive patients who were free of stroke or had no history of stroke were considered as controls. Case with less than three follow-ups for hypertension treatment before the first stroke attack and controls with less than three follow-ups for hypertension treatment were excluded. Patients whose medical card had incomplete information were also excluded from the study.

### Sample size determination and sampling technique

Sample size was determined using Epi-Info version 7 for unmatched case-control study based on the following assumption; 95% confidence level, power = 80%, adjusted odds ratio = 2.47, proportion of controls with missed medical follow up appointment,12.9%, for cases,26.8% [22] and with 2:1 controls to case ratio. Considering 5% non-response rate, the final sample size was determined to be 326 (109 cases and 217 controls). There were 13 public hospitals found in Addis Ababa. Ten of them were providing follow-up service for hypertensive patients. Six hospitals were selected using the lottery method. Proportional allocation to each selected hospital was done by calculating sample fractions based on last year’s one-month record data of hypertensive patients. All stroke cases were included in the study during the study period while two comparable controls were recruited consecutively after each stroke case was enrolled. Controls were selected from the respective hospitals that yielded the cases.

### Data collection tool and techniques

Questionnaire was adapted and from various studies and contextualized based on study objective [15, 18, 19, 21, 22, 30–35]. The questionnaire was prepared in English version and translated into local language Afaan Oromo and Amharic and, then translated back to English by language experts to check for consistency. The quality of the questionnaire was assured by properly designing and pre-testing the tool, and training the data collectors and supervisors before the actual data collection for two days. The questionnaire was pretested on 5% of sample size at Alert referral hospital and necessary modifications were made on some items of the questionnaire before the actual data collection. Data collectors waited until the study unit physician complete their consultation. After they finished, cases and controls were identified from the medical record history of patients. After cases and controls were differentiated, data was collected by using a structured questionnaire and checklist. Six trained BSc nurses were recruited to collect data and supervised by 3 General practitioners. The tools have three parts. The first part is about socio-demographic characteristics of participants. The second part is asks about behavioural factors like smoking cigarettes, alcohol drinking, chat chewing, salty diets intake, fatty diets consuming, physical exercise, frequency of follow-up appointments, medical follow-up, medication adherence status. Medication adherence status was assessed by using Mo risky medication adherence 8 point scale which is a self-reporting tool. It has eight questions, seven of which are closed dichotomous (yes/no) and one of which is Liker-scale question. Each item measures specific adherence behaviour. The score obtained from the sum of all correct answers was used to determine the degree of adherence. The internal consistency of items was found to have 0.76 alpha coefficients. The third part is clinical factor which was taken from medical records like duration since diagnosed of hypertension, total cholesterol level, uncontrolled systolic blood pressure, uncontrolled diastolic blood pressure, low high density lipoprotein, high low density lipoprotein, triglycerides, and diabetic mellitus.

### Operational definition

**Medication adherence.** Medication adherence was measured using the Mo risky 8-point scale for antihypertensive drugs. Adherent if they score > 6 points and non-adherent if they score of ≤ 6 points [33–36].

**Physical exercise.** Practiced regular physical exercise if respondents reported that they exercise moderate physical activities for ≥30 minutes per day for at least 5 days per week. Moderate physical exercise means walking, Jogging, cycling, guarding and household chores [25, 37, 38].

**Cigarette smoker.** Ever smoker:—respondents who reported that they had been ever smoking cigarette or any tobacco products.

**Current smokers.** Respondents who reported that they were smoking cigarette/any tobacco products after diagnosis of hypertension [25, 37].

**Alcohol drinker.** Ever drinker:—respondents who reported that they had been ever drinking any alcohol before diagnosis of hypertension.

**Current drinker.** Respondents who reported that they were drinking > 2 units of alcohol for male; >1 units of alcohol for female after diagnosis of hypertension [25, 37]. Total cholesterol level **norma**l if < 200 mg/dl and **high** if ≥ 200 mg/dl, low density lipoprotein **high** if ≥ 100 mg/ dl, and serum triglyceride level **high** if ≥ 150 mg/dl [38]. Systolic blood pressure **controlled** if <140 mmHg and **uncontrolled** if ≥140 mmHg, diastolic blood pressure **controlled** if < 90 mmHg and **uncontrolled** if ≥ 90 mmHg [38].

### Data analysis

Data were coded and entered in to Epi-data v.3.1 and exported to SPSS v.23 for analysis. Descriptive statistics were computed for independent variables. Multi-collinearity among the independent variables was assessed by using variance inflation factors. However, no significant multi-collinearity was detected as the variance inflation factor for all variables was less than five. Bi-variable logistic regression analysis was conducted to see the association of each independent variable to the outcome variable and variables with p-value<0.25 were identified and fitted to the multivariable logistic regression analysis through the backward stepwise method to reduce the effects of confounders and to identify the independent effects of each variable on the outcome variable. The Hosmer-Lemeshow goodness of fit test was used to assess the model fitness. Finally, adjusted odds ratio with 95% confidence interval and p-value<0.05 were reported to declare significant determinants of stroke.

### Ethics statement

The ethical clearance was obtained from the Institutional Review Board (IRB) of Ambo University College of Medicine and Health Science and ethical committee with the reference number of PGC/208/2021. Then officials at different levels of the selected hospitals were communicated through cooperation letter written from the post graduate coordinator of Ambo University. The responsible bodies at each hospital were informed about the purpose of the study. Informed written consent was obtained from all subjects for their participation after the nature of the study is fully explained to them. Only those who gave consent have participated in the study. Throughout the study, participants were informed that data was kept private and confidential and used only for research purposes. The participants also assured that they have the right to refuse or withdraw if they are not comfortable with questions at any time as their participation is voluntary and the failure to participate in the study as it did not result in a penalty.

## Results

### Socio demographic characteristics of the study participants

A total of 326; 109 cases and 217 controls participants were involved in the study yielding response rate of 100%. Sixty two (56.9%) of the cases and 116 (53.5%) of the controls were male. The mean age of the cases and controls was 52.3(SD ±13.8) and 53.2(SD ± 14.6) years, respectively (Table 1).

**Table 1:** Socio demographic characteristics of the stroke patients who were on follow-up in public hospitals of Addis Ababa, Ethiopia (n = 326), 2021.

Variables	Cases(n = 109)	Controls(n = 217)
	N (%)	N (%)
Sex		
Male	62(56.9)	116(53.5)
Female	47(43.1)	101(46.5)
Age (in years)		
< 45	37(33.9)	69(31.8)
45–64	52(47.7)	82(37.8)
≥65	20(18.3)	66(30.4)
Religion		
Orthodox	74(67.9)	165(76.0)
Protestant	21(19.3)	31(14.3)
Muslim	9(8.3)	15(6.9)
Others	5(4.6)	6(2.8)
Educational level		
No formal education	24(22.0)	49(22.6)
Primary school	32(29.4)	68(31.3)
Secondary school	30(27.5)	43(19.8)
College +	23(21.1)	57()26.3)
Occupation		
Farmer	11(10.1)	21(9.7)
Daily labourer	14(12.8)	18(8.3)
Government employee	17(15.6)	41(18.9)
Self- employee	8(7.3)	19(8.8)
Non-government employee	20(18.3)	47(21.7)
Housewives	35(32.1)	61(28.1)
Others	4(3.7)	10(4.6)
Marital status		
Single	14(12.8)	30(13.8)
Married	67(65.1)	144(66.4)
Divorced	6(5.5)	13(6.0)
Widowed	22(20.2)	30(13.8)
Residence		
Urban	95(87.2)	191(88.0)
Rural	14(12.8)	26(12.0)

Others- Waqefata, Catholic

### Behavioral characteristics of participants

Among the study participants, 45 (41.3%) of the cases and 17 (7.8%) of the controls were current cigarette smokers. Forty (36.7%) of the cases and 63 (29.0%) of the controls had missed their follow-up appointment (Table 2).

**Table 2:** Behavioral characteristics of the study participants.

Variables	Cases(n = 109)	Controls(n = 217)
	N (%)	N (%)
Ever cigarette smokers		
No	55(50.5)	119(54.8)
Yes	54(49.5)	98(45.2)
Current cigarette smokers		
No	64(58.7)	200(92.2)
Yes	45(41.3)	17(7.8)
Ever alcohol drinkers		
No	39(35.8)	91(41.9)
Yes	70(64.2)	126(58.1)
Current alcohol drinkers		
No	75(68.8)	189(87.1)
Yes	34(31.2)	28(12.9)
Ever chat chewers		
No	58(53.2)	117(53.9)
Yes	51(46.8)	100(46.1)
Current chat chewers		
No	62(56.9)	133(61.3)
Yes	47(43.1)	84(38.7)
Reduce salt consumption		
No	37(33.9)	84(38.7)
Yes	72(66.1)	133(61.3)
Reduce consuming fatty diet		
No	51(46.8)	89 (41.0)
Yes	58(53.2)	128(59.0)
Regular physical exercise		
Not practiced	67(61.5)	99(45.6)
Practiced	42(38.5)	118 (54.4)
Frequency of follow up		
Every Month	16(14.7)	11 (5.1)
Every 2 month	7(6.4)	27(12.4)
Every 3 month +	86(78.9)	179(82.5)
Missed follow up		
No	69(63.3)	154(71.0)
Yes	40(36.7)	63(29.0)
Medication adherence		
Non- adherent	50(45.9)	47(21.7)
Adherent	59(54.1)	170(78.3)

The MMAS-8 Scale, content, name, and trademarks are protected by US copyright and trademark laws. Permission for use of the scale and its coding is required. A license agreement is available from MMAR, LLC., www.moriskyscale.com

### Clinical characteristics of participants

The mean systolic blood pressure was 162(SD ±17) in the cases and 148 (SD ± 16) in the controls. Of a total of 326 participants, 59(54.1%) of the cases and 104 (47.9%) of the controls were 5 years and older since they were diagnosed with hypertension. The majority, 88(80.7%) of the cases and 130(59.9%) of the controls, had uncontrolled systolic blood pressure (Table 3).

**Table 3:** Clinical factors of the study participants.

Variables	Cases(n = 109)	Controls(n = 217)
	N (%)	N (%)
Duration since diagnosis		
< 5 years	50(45.9)	113(52.1)
≥5 years	59(54.1)	104(47.9)
Systolic blood pressure		
Controlled	21(19.3)	87(40.1)
Uncontrolled	88(80.7)	130(59.9)
Diastolic blood pressure		
Controlled	20(18.3)	96(44.2)
Uncontrolled	89(81.7)	121(55.8)
Total cholesterol		
Normal	42(38.5)	153(70.5)
High level	67(61.5)	64(29.5)
High density lipoprotein(HDL)		
Normal	70(64.2)	128(58.1)
Low Level	39(35.8)	91(41.9)
Low density lipoprotein(LDL)		
Normal	32(29.4)	156(71.9)
High level	77(70.6)	61 (28.1)
Triglycerides		
Normal	67(61.5)	166(76.5)
High level	42(38.5)	51(23.5)
Diabetic mellitus		
No	66(60.6)	190(87.6)
Yes	43(39.4)	27(12.4)
Family history of stroke		
No	86(78.9)	181(83.4)
Yes	23(21.1)	36(16.6)

### Types of stroke and diagnostic tools performed

Out of 109 cases, 60(55%) were ischemic stroke and 49(45%) were haemorrhagic stroke. Eight five (78%) of cases were diagnosed by CT-scan, 18(16.5%) by MRI and 6(5.5%) clinically.

### Determinants of stroke

Bi-variable logistic regression analysis showed that 13 variables were associated with stroke. After adjustments for possible effects of confounding variables, current cigarette smokers, current alcohol drinkers, medication non adherence, uncontrolled systolic blood pressure, uncontrolled diastolic blood pressure, high low density lipoprotein and diabetic mellitus was identified to be determinants of stroke (Table 4).

**Table 4:** Bi-variable and multi-variable logistic regression analysis of determinants of stroke among adult hypertensive patients on follow up in Addis Ababa public hospitals (N = 326), 2021.

Variables	Cases (n = 109)	Controls (n = 217)	COR(95%CI)	AOR(95%CI)
Age				
< 45	37(33.9)	69(31.8)	1	1
45–64	52(47.7)	82(37.8)	1.18(0.69, 2.01)	0.87(0.40, 1.88)
≥65	20(18.3)	66(30.4)	0.57(0.29, 1.1)	0.49(0.15, 1.66)
Current cigarette smoker				
No	64(58.7)	200(92.2)	1	1
Yes	45(41.3)	17(7.8)	8.27(4.43, 15.4)	5.5(2.48,12.4)*
Current alcohol drinker				
No	75(68.8)	189(87.1)	1	1
Yes	34(31.2)	28(12.9)	3.06(1.74, 5.39)	4.27(1.94, 9.3)*
Regular physical exercise				
Practiced	42(38.5)	118(54.4)	1	1
Not practiced	67(61.5)	99(45.6)	1.90(1.19, 3.04)	1.65(0.88, 3.13)
Frequency of follow up				
Monthly	16(14.7)	11(5.1)	1	1
Every 2 month	7(6.4)	27(12.4)	0.18(0.06, 0.56)	0.20(0.04, 1.01)
Every 3 month +	86(78.9)	179(82.5)	0.33(0.15, 0.74)	0.48(0.14, 1.62)
Missed follow up appoint				
No	69(63.3)	154(71.0)	1	1
Yes	40(36.7)	63(29.0)	1.42(0.87, 2.31)	1.33(0.64, 2.76)
Medication adherence				
Non-adherent	50(45.9)	47(21.7)	3.07(1.87, 5.04)	3.2(1.62, 6.44)*
Adherent	59(54.1)	170(78.3)	1	1
Systolic blood pressure				
Controlled	21(19.3)	87(40.1)	1	1
Uncontrolled	88(80.7)	130(59.9)	2.80(1.62, 4.85)	3.4(1.64, 7.16)*
Diastolic blood pressure				
Controlled	20(18.3)	96(44.2)	1	1
Uncontrolled	89(81.7)	121(55.8)	3.53(2.03, 6.1)	4.2(2.06, 8.93)*
Total cholesterol level				
Normal	42(38.5)	153(70.5)	1	1
High level	67(61.5)	64(29.5)	3.86(2.35, 6.19)	1.41(0.70, 2.84)
Low density lipoprotein				
Normal	32(29.4)	156(71.9)	1	1
High level	77(70.6)	61(28.1)	6.15(3.71, 10.2)	6.89(3.57, 13.4)*
Triglycerides				
Normal	67(61.5)	166(76.6)	1	1
High level	42(38.5)	51(23.5)	2.04(1.24, 3.36)	1.11(0.55, 2.24)
Diabetic mellitus				
No	66(60.4)	190(86.6)	1	1
Yes	43(39.4)	27(12.4)	3.07(1.91, 5.81)	3.25(1.58, 6.69)*

* Significant at p< 0.05, AOR-Adjusted Odds Ratio CI- confidence interval 1-reference

## Discussion

Stroke ‘sudden death of brain cells due to insufficient oxygen supply’ is the leading cause of morbidity and mortality, and it can lead to permanent disabilities on patients, imposing a financial burden on their families, communities, and the country as a whole. Hence, early detection and control of determinant factors is crucial to avoid the development of stroke and help in decreasing overall adult mortality and morbidity. This study attempted to identify the determinants of stroke among adult hypertensive patients on follow up in public hospitals of Addis Ababa, Ethiopia.

In this study, hypertensive patients those who currently smoke cigarette were 5.55 times more likely to develop stroke than non-smokers.

This finding is consistent with studies conducted in Tanzania, Ghana and Nigeria (SIREN) and Finland [18, 19, 21]. The possible explanation might be due to tobacco products contain addictive and harmful chemical substances that are absorbed into the bloodstream, which may predispose blood vessels to thrombosis, atherosclerosis, and facilitate platelet aggregation by causing an imbalance between brain vascular coagulation and abnormal fibrinolysis. This further affect blood-brain barrier’s function and disrupt normal endothelial cells, which results in stroke [39]. This finding dis-agree with study conducted in Tigray Ethiopia [22].

The study found that, hypertensive patient who currently drink alcohol were 4.27 times more likely to develop stroke than their counterparts. Our finding was consistent with study conducted in Tigray Ethiopia and Nigeria (INTERSTROKE) [15, 22, 31]. Alcohol consumption can also harm the liver by preventing it from producing substances that aid in blood clotting. This can raise chances of getting a stroke due to brain haemorrhage [40].

Hypertensive patients who were not adherent to their medication were 3.23 times higher to develop stroke compared to those who were adherent to their prescribed at hypertensive medication. This finding is comparable with studies done at Tigray and northern Nigeria [17, 22]. The possible explanation might be due to different factors which contribute to patients’ non-adherence was due to nature of treatment like complexity of the regimen and adverse effects, characteristics of the patients; forgetfulness, inability to finish as they feel better, lack of understanding the prescription, inability to pay prescription charges, attitudes of patients toward treatment, patient–doctor relation [37]. Patients with hypertension who do not take their medication as prescribed may worsen their condition and make blood pressure uncontrollable resulting increasing odds of developing stroke [41].

Failure to control both systolic and diastolic blood pressure during course of treatment was one of the main determinant factors of stroke in this study. Hypertensive patients with uncontrolled systolic blood pressure were 3.42 times more likely to develop stroke compared to their counterparts. Similarly, hypertensive patients with uncontrolled diastolic blood pressure were 4.29 times higher odds of developing stroke compared to their counterparts. This finding is in line with studies conducted in Tigray, Nigeria, Tanzania and Thailand [19, 22, 30, 31]. The possible explanation of this might be uncontrolled systolic and diastolic blood pressure were developed due to lack of appropriate treatment, unhealthy lifestyle, certain medical conditions like diabetics, smoking, heavy alcohol consumption, and adherence problem [39].

Hypertension predisposes to atherosclerotic plaque formations as well as micro-aneurysms in the brain. Uncontrolled blood pressure damage the arteries and form weak places that rupture easily (micro-aneurysmal) and plaque accident results increased risk of stroke [16].

Moreover, odds of stroke among hypertensive patients those who had high low density lipoprotein cholesterol level were 6.89 folds higher compared to their counterparts. This finding is consistent with studies done in Tigray and Saud Arabia [22, 32]. Low density lipoprotein carries cholesterol to cells and deposits excess cholesterol in arteries, reducing blood and oxygen supply to the brain and therefore reducing lipid profile can reduce atherosclerotic plaques, which results in a decreased risk of stroke [42]. But, study conducted in Finland revealed that there was no statistically significant differences among the groups with respect to low density lipoprotein variation [18]. This might be due to sample size, demographic difference and economic status.

Similarly, odds of developing stroke among hypertensive patients having diabetic mellitus were 2.25 times higher compared to those hypertensive patients with non-diabetic. This finding is consistent with studies done in Nigeria, Tanzania and Saudi Arabia [19, 31, 32]. This is due to the fact that, diabetes mellitus causes various micro and macro-vascular changes often ending with major clinical complications [43]. It is one of the risk factor for development of atherosclerosis and the excess risk of stroke [39].

### Strength and limitation of the study

The strength of this study is that cases and controls were selected from the same source population (controls were selected from the respective hospitals where the cases came from). As a result, cases and controls were comparable. Blood pressure was obtained from review of the patient’s chart, so information on how blood pressure was measured was not available. Because some of our data were extracted from patient records, we could not get complete data from the patients’ medical records on some important variables such as height and weight to calculate body mass index and hence we could not be assessed it. Because it was confined to only public hospitals, hypertensive patients having enough money might be followed in a private hospital and higher clinics and the study unfortunately did not assessed such patients. Despite the authors’ efforts to create representative sample by carefully identifying the study population and choosing the appropriate comparison, recall bias and social desirability bias can still occur.

## Conclusion and recommendation

In this study; Cigarette smoking, alcohol use, medication non-adherence, uncontrolled systolic and diastolic blood pressure, high-low-density lipoprotein, and diabetic mellitus have been identified as determinants of stroke. Therefore, future intervention and prevention approaches should focus on these determinants.

## Supporting information

S1 File (DOCX)

S2 File (DOCX)

S3 File (DOCX)

S4 File (SAV)

## List of abbreviations

AAH: Addis Ababa Hospitals
CT-: Computed Tomography
DALYs: Disability Adjusted Life Years
MRI-: Magnetic Resonance Imaging
NCD: Non-Communicable Disease
SPSS: Statistical Package for the Social Science
WHO: World Health Organization
