# Supplementary material for: Determinants of stroke among adult hypertensive patients on follow up in Addis Ababa public hospitals, Ethiopia: A case control study
Source: PLoS One. 2024 Sep 3;19(9):e0286845. doi: 10.1371/journal.pone.0286845 (PMC11371249; doi:10.1371/journal.pone.0286845)
Supplement: S1 File — (DOCX) [file pone.0286845.s001.docx]

# **Appendix**

Ambo University

College of Medicine and Health Sciences

Department of Public Health in Epidemiology

Determinants of stroke among adult hypertensive patients on follow up in public hospitals of Addis Ababa, Addis Ababa, Ethiopia, 2021

## **Appendix A. Information sheet**

Good morning/good afternoon sir! My name is ____________________________ I am working as data collector for the study being conducted at this health facility by **Feyisa Teshome Temesgen** who is studying for his master’s degree at Ambo University, College of Medicine and Health Science. I kindly request you to give me your attention to explain you about the study and being selected as the study participant.

**Study title:**

Determinants of stroke among adult hypertensive patients on follow up in public hospitals of Addis Ababa, Addis Ababa, Ethiopia, 2021

**Study purpose:**

This study will assess the determinants of stroke among adult hypertensive patients on follow in Addis Ababa.

**Procedure and duration:**

I will interview clients using a questionnaire to get pertinent data that is helpful for the study. There are about few questions to answer where I will fill the questionnaire by interviewing clients. The interview will take about 30 minute.

**Risks and benefit of the study:**

No risk of being participating in this study, but only taking few minutes from patient’s time. There will not be any direct payment for participating in this study. But the findings from this study may reveal important information for the local health planners.

**Confidentiality:**

The information gathered from this study will not be disclosed to others. The hard copy will be kept confidential. The data in computer will be coded not to include their name and specific identifier of the client.

**Rights:**

Participation in our study is fully voluntary and under written consent. Considering the importance of the study to your health facility, you are free to decide on it. If any violation of ethical rules and conduct is seen throughout study, your health facility has full right to withdraw and stop study at any time.

Persons to contact: If you have any question to ask, please contact

Principal investigator:

Dureti Tirfessa

Phone: 0965025908

Email: duretitirfessa222@gmail.com

Are you satisfied with the information provided so far?

1. Yes………………………….. Continue to the next page

2. No …………………………… I won’t participate

## **Appendix B. Voluntary Consent form**

I (the respondent), the undersigned, am told that the researcher is going to conduct study in this hospital to identify the determinants of stroke among hypertensive, and s/he aware me the first time s/he meets the information gathered will be used to know determinants of stroke in those hospitals.

Procedures to be followed: Participation in this study requires I ask you some questions which I will record the answers in the questionnaire. You have the right to refuse to participate in this study. You will receive the same care whether you agree to join the study or not and your decision will not change the care you will receive from the facility today/later or that you will get from any other facility at any other time. Participation is voluntary and you may ask questions related to the study at any time. You may refuse to respond to any question and you may also stop the interview at any time without any consequences to the services you receive from this health facility or any other organization now or in the future.

Do you agree? Yes No

Signature of participant: __________ Date___________________________

Data collector’s Signature: ______________________Date___________________________

Supervisor’s Signature: ________________________ Date___________________________

Signature of participant: __________

Thank you for your cooperation’s!

## **Appendix C. English Version Questionnaire**

| **Part 1.** Socio demographic characteristics Instructions: the following questions are about socio demographic characteristics of the study participant please circle on the numbers under the coding categories or record on the space provided according to the participants response | | | | | | | | | | |
| --- | --- | --- | --- | --- | --- | --- | --- | --- | --- | --- |
| S. No | | | | Questions | | Alternative choice for respondents | | | Skip to Q-- | |
| 101 | | | | Sex | | 1. Male 2. Female | | |  | |
| 102 | | | | Age | | _____ years | | |  | |
| 103 | | | | Religion | | 1. Orthodox 2. Protestant 3. Muslim 4. Catholic 5. Wakefata 6. Others_________ | | |  | |
| 104 | | | | Educational status | | 1. No formal education 2. Primary school(1-8) 3. Secondary school(9-12) 4. College and above | | |  | |
| 105 | | | | Occupational status | | \| 1. Farmer 2. Daily laborer 3. Government employee 4. Self-employee 5. Non-Government employee 6. Student 7. Housewives 8. Other(specify)_____ \| \| --- \| | | |  | |
| 106 | | | | Marital status | | 1. Single 2. Married 3. Divorced 4. Widowed | | |  | |
| 107 | | | | Place of residence | | 1. Urban 2. Rural | | |  | |
| **Part 2 Behavioral related questions** | | | | | | | | | | |
| 201. | | | | | Have you ever smoke cigarette? | 1. Yes 2. No | | | No skip to, Q, 203. | |
| 202. | | | | | Do you smoke cigarette currently after diagnosis of hypertension? | 1. Yes 2. No | | |  | |
| 203 | | | | | Have you ever drink Alcohol? | 1. Yes 2. No | | | No skip to Q,207 | |
| 204 | | | | | Do you drink alcohol currently after diagnosis of hypertension? | 1. Yes 2. No | | | No skip to Q,207 | |
| 205 | | | | | Type of alcohol you drink (multiple response is possible probe for further responses, circle all that applies ) | 1. Beer 2. Wine 3. “Farso” 4. “Tej” 5. “Araqe(Oz)” 6. Others_______ | | |  | |
| 206 | | | | | Average amount of alcohol you drink/day(in bottle, glass, cup, Oz | ________________  __________________  __________________ | | |  | |
| 207 | | | | | Have you ever chew chat? | 1. Yes 2. No | | | No skip to Q,209 | |
| 208 | | | | | Do you chewing chat currently after you diagnosis of hypertension? | 1. Yes 2. No | | |  | |
| 209 | | | | | Do you consume salt by reducing the amount your families consumed? | 1. Yes 2. No | | |  | |
| 210 | | | | | Do you reduce eating diet high in fat such as Fatty meal and animal product? (Meat, egg, cheese, butter, fish, cream)? | 1. Yes 2. No | | |  | |
| 211 | | | | | Do you perform physical exercise? | 1. Yes 2. No | | | No skip to Q,216 | |
| 213 | | | | | If you say yes for q211, what type of physical exercise you perform? (multiple response is possible probe for further responses, circle all that applies ) | 1. Walking 2. Jogging 3. Cycling 4. Household chores 5. Others­­­­­­­­_____________ | | |  | |
| 214 | | | | | How many days you exercise in a week? | _____________ | | |  | |
| 215 | | | | | For how long do you exercise per days? | In hours/minute___________ | | |  | |
| 216 | | | | | How often is your follow-up? | 1. Monthly 2. Every 2 month 3. Every 3 month 4. Other specify | | |  | |
| 217 | | | | | Have you ever missed your follow-up appointment (as per the health professionals appoint you)? | 1. Yes 2. No | | |  | |
| **Part 2. 1 Morisky Medication adherence standard checklist** | | | | | | | | | | |
| Question | | | | | | | | | Yes | No |
| 301 | \| Do you sometimes forget to take your pills? \| \| --- \| | | | | | | | |  |  |
| 302 | People sometimes miss taking their medications for reasons other than forgetting. Thinking over the past two weeks, were there any days when you did not take your medicine? | | | | | | | |  |  |
| 303 | Have you ever cut back or stopped taking your medicine without telling your doctor because you felt worse when you took it? | | | | | | | |  |  |
| 304 | When you travel or leave home, do you sometimes forget to bring along your medicine? | | | | | | | |  |  |
| 305 | Did you take all your medicine yesterday? | | | | | | | |  |  |
| 306 | When you feel like your symptoms are under control, do you sometimes stop taking your medicine? | | | | | | | |  |  |
| 307 | Taking medicine every day is a real inconvenience for some people. Do you ever feel hassled about sticking to your treatment plan? | | | | | | | |  |  |
| 308 | How often do you have difficulty remembering to take all your medicine**? Please circle the correct answer** | | | | | | | | 4. Never/rarely 3. Once in a while  2.Sometimes  1. Usually  0. All the time | |
| **Part 3. Clinical factors** | | | | | | | | | | |
| 401 | | How long has it been since you were diagnosed with hypertension? | | | | | | ______________ | | |
| 402 | | Do you have family history of stroke? | | | | | | 1.Yes  2. No | | |
| **Data abstraction format from medical record** | | | | | | | | | | |
| 403 | | | Type of stroke | | | | 1. Ischemic stroke 2. Hemorrhagic stroke | | | |
| 404. | | | Methods performed to diagnose stroke | | | | 1. CT-scan 2. MRI 3. Clinical | | | |
| 405 | | | Blood Pressure during first attack of stroke for cases or during data collection for controls | | | | - 1. __________mmHg (during the first attack of stroke or during the data collection for controls. | | | |
| 406 | | | Blood Pressure before attacked of stroke for cases or before data collection for controls | | | | - 1. ________mmHg (before attacked of stroke(cases) or before data collection for (controls) patients | | | |
| 407 | | | Laboratory results | | | | 1. Total cholesterol______________ 2. High density lipoprotein________ 3. Low density lipoprotein________ 4. Triglycerides________________ | | | |
| 408 | | | Diabetic Mellitus | | | | 1. No 2. Yes | | | |
