# Supplementary material for: Determinants of stroke among adult hypertensive patients on follow up in Addis Ababa public hospitals, Ethiopia: A case control study
Source: PLoS One. 2024 Sep 3;19(9):e0286845. doi: 10.1371/journal.pone.0286845 (PMC11371249; doi:10.1371/journal.pone.0286845)
Supplement: S2 File — (DOCX) [file pone.0286845.s002.docx]

**Information sheet Amharic version**

**በአምቦ ዩኒቨርሲቲ ህክምናና ጤና ሳይንስ ኮሌጅ የድህረ ምረቃ መርሃግብር (ከእንግሊዝኛ የተተረጎመ)**

**የተከበሩ የጥናቱ ተሳታፊ!**

እንዴትአደራችሁ/ዋላችሁ፡ እኔ ስሜ _______________________________የተባልኩት **ፈይሳ ተሾመ**  በአምቦ ዩኒቨርሲቲ የሁለተኛ ዲግሪ/ማስተርስ ተማሪ ሲሆን በጤና ተቋማት ላይ እያካሄደ ላለው ጥናት በመረጃ ሰብሳቢነት ነው የምሰራው፡፡ እርስዎም የጥናቱ ተሳታፊ ሆነው ስለተመረጡ ስለጥናቱ አጠቃላይ ሁኔታ ገለጻ እንዳደርግልዎት እንዲተባበሩኝ በትህትና እጠይቃለሁ፡፡

**የጥናቱዓላማ፡** ይህ ጥናት የሚያተኩረው በአዲስ አበባ የመንግስት ሆስፒታሎች የሚከታተሉ የደም ግፊት ተመላላሽ ታካሚዎችን ለስትሮክ (ነርቭ በሽታ) የሚዳርጓቸው ዋና ዋና ነገሮችን ማጥናት ላይ ነው፡

**የጥናቱ ተሳታፊዎች ማንነት፡** በአዲስ አበባ በሚገኙ የመንግስት ሆስፒታሎች የሚከታተሉ የደም ግፊትና ስትሮክ/ነርቭ በሽታ ታካሚዎች በዚህ ጥናት ውስጥ ይሳተፋሉ፡፡

**በመሳተፍዎ የሚያገኙት ጥቅም እና ጉዳት፡**በጥናቱ ስለ ተሳተፉ ቀጥተኛ የሆነ ገንዘብም ሆነ ሌላ ጥቅም አያገኙም፡፡ ነገር ግን የእርስዎ ድምጽ የደም ግፊት ታካሚዎችን ለስትሮክ/ለነርቭ በሽታ የሚዳርጓቸው ዋና ዋና ነገሮችን እንድናውቅ ይረዳናል በዚሁም መሰረት ለሚመለከተዉ አካል አስፈላጊዉ መልዕክት እነዲተላለፍና ማስተካከያ እንዲደረግ ይደረጋል፡፡ በሌላ መልኩ በጥናቱ ስለተሳተፉ ቢበዛ 30 ደቂቃ ከመስጠት ዉጭ ምንም አይነት የአካል ወያም የስነ ልቦና ጉዳት አይደርስብዎትም፡፡

**የመረጃን ምስጢር መጠበቅ፡** የእርስዎ ስም በመጠይቁ ወረቀት ላይ አይፃፍም፡፡የሚሰጡን መረጃ በምንም መልኩ ለሶስተኛ ወገን አይታይም፡፡ በጥናቱ ዉስጥ የመሳተፍም ያለመሳተፍም እንዲሁም በፈለግዎት ጊዜ የማቋረጥ መብትዎ የተጠበቀ ነው፡፡ስለ ጥናቱ ማንኛውም ዓይነት ጥያቄ ቢኖርዎት ወይም ስለጥናቱ የመጨረሻ ውጤት ማወቅ ቢያስፈልግዎት በሚከተለው የጥናቱ ባለቤት አድራሻ ማግኘት ይችላሉ፡

**የጥናቱ ባለቤት አድራሻ**

Feyisa Teshome Temesgen.

ስልክ: +251-921406387, ኢሜል = [fteshome3@gmail.com](mailto:fteshome3@gmail.com)

ባገኙት መረጃ እረክተዋል/ደስተኛ ነዎት?

1. አዎ………………………….. ወደቀጣዩ ገጽ ይቀጥሉ

2. አይደለሁም …………………………መሳተፍ አልፈልግም

**Informed consent form Amharic version**

**ስምምነት ቅጽ**

እኔ ከዚህ በታች የምፈርመው ግለሰብ በአዲስ አበባ የመንግስት ሆስፒታሎች የሚከታተሉ የደም ግፊት ተመላላሽ ታካሚዎችን ለስትሮክ (ነርቭ በሽታ) የሚዳርጓቸው ዋና ዋና ነገሮችን ለማወቅ በሚጠናው ጥናት ውስጥ ተሳታፊ እንድሆን መስማማቴን እየገለጽኩ ጥናቱ በፈቃደኝነት ላይ የተመሰረተ መሆኑንም ተረድቻለሁ፡፡ከዚህ ቀጥሎ በሚገኘው መጠይቅ የምሰጠው መረጃም ሚስጢርነቱ የተጠበቀ እንደሚሆንም በሚገባ ተነግሮኛል፡፡በጥናቱ ውስጥ ተሳታፊ መሆኔም አለመሆኔም በግል ሕይወቴ ውስጥ ችግር እንደማያመጣብኝም ተነግሮኛል፡፡በመጨረሻም ስለጥናቱ እና የጥናቱ ተሳታፊ እንደመሆኔ ባለኝ መብት ዙሪያ ጥያቄ ቢኖረኝ ፈይሳ ተሾመ ተባለውን የጥናቱ ዋና ባለቤት ማነጋገር አንደምችልም ተረድቻለሁ፡፡

የተሳታፊው ፊርማ_________________________________ ቀን_____________________________

የመረጃ ሰብሳቢው ፊርማ___________________________ ቀን_______________________

**Appendix D. Amharic Version Questionnaire**

| **ክፍል 1** **።** የታማሚ የ ኢኮኖሚ እና የማህበራዊ ሁኔታን የተመለከቱ ከዚህ በታች ያሉት ጥያቄዎች ስለ ታማሚው ማንነት አንዳንድ ነገሮችን ይጠይቃል፡፡ በመለያ ስር ያለውን ቁጥር የጥናቱ ተሳታፊ ምላሽ ሲሰጥ ይከበብ፡፡እንዲሁም ክፍት በተተዉት ቦታዎች የታማሚውን ቀጥታ መልስ ይመዝገብ፡፡ | | | | | | | | | | |
| --- | --- | --- | --- | --- | --- | --- | --- | --- | --- | --- |
| ተራ.ቁ | | ጥያቄዎች | | ምላሽና የኮድ መደብ | | | | ይዝለሉት | | |
| 101. | | ፆታ | | ሀ. ወንድ  ለ. ሴት | | | |  | | |
| 102 | | እድሜ? (በሙሉ አመት ይቀመጥ) | | ____________ | | | |  | | |
| 103. | | ሃይማኖት | | ሀ. ኦርቶዶክስ  ለ. ፕሮቴስታንት  ሐ. ሙስሊም  መ. ካቶሊክ  ሠ. ወቄፈታ  ረ. ሌሎች-------------- | | | |  | | |
| 104 | | የተማሩት ከፍተኛው የትምህርት ደረጃ? | | ሀ. ትምህርት ቤት ጋብቶ/ታ ያልተማረ/ች  ለ. የመጀመሪያ ደረጃ(1-8)  ሐ. ሁለተኛ ደረጃ(9-12)  መ. ኮሌጅ እና በላይ | | | |  | | |
| 105 | | በአሁኑ ሰዓት መደበኛ ስራዎ ምንድነው? | | \| ሀ. አርሶ አደር  ለ. የቀን ሰራተኛ  ሐ. የመንግስት  መ. የግል ስራ  ሠ. የመንግስት ያልሆነ ድርጅት  ረ. ተማሪ  ሰ. የቤት እመቤት  ሸ. ሌሎች----------- \| \| --- \| | | | |  | | |
| 106. | | አሁን ያለዎት የጋብቻ ሁኔታ ምንድነው? | | ሀ. ያላገባ/ች  ለ. ያገባ/ች  ሐ. የፈታ/ች  መ. የሞተበት/ ችባት | | | |  | | |
| 107 | | የመኖሪያ ቦታ የት ነው? | | ሀ. ከተማ  ለ. ገጠር | | | |  | | |
| **ክፍል 2። ከ ፀባይ ጋር የተያያዙ ጥያቄዎች** | | | | | | | | | | |
| 201. | | ስጋራ አጭሰው ያውቃሉ? | | ሀ. አዎ  ለ. አይ | | | መልስ አይ ከሆነ ወደ ጥ/ቁ 203, ይለፉ | | | |
| 202. | | መልስ አዎ ከሆነ ጥ/ቁ 201፣ የደም ግፊት እንዳለብዎት ካወቁ በኋላ ስጋራ አጭሰው ያውቃሉ? | | ሀ. አዎ  ለ. አይ | | |  | | | |
| 203 | | አልኮል ጠጥተው ያውቃሉ? | | ሀ. አዎ  ለ. አይ | | | መልስ አይ ከሆነ ወደ 207 ጥያቄ ይለፉ | | | |
| 204 | | መልስ አዎ ከሆነ ጥ/ቁ 203 የደም ግፊት እንዳለብዎት ካወቁ በኋላ አልኮል ጠጥተው ያውቃሉ? | | ሀ. አዎ  ለ. አይ | | | መልስ አይ ከሆነ ወደ 207 ጥያቄ ይለፉ | | | |
| 205 | | መልስ አዎ ከሆነ ጥ/ቁ 204 የምትጠጡት የአልኮል ዓይነት? (ከአንድ በላይ መልስ መስጠት ይቻላል) | | ሀ. ቢራ  ለ. ወይን  ሐ. “ጣላ”  መ.“ጠጅ”  ሠ.“አራቄ/ካትካላ”  ረ. ሌላ ካለ ይገለጽ_____________ | | |  | | | |
| 206 | | በቀን የሚጠጡት የአልኮል መጠን በአማካይ ምን ያህል ነው? (ባጠርሙስ፣ብርጭቆ ፣ ኩባያ ፣ “መለኪያ”? | | ________________  __________________  __________________ | | |  | | | |
| 207 | | ጫት ቅመው ያውቃሉ | | ሀ. አዎ  ለ. አይ | | | መልስ አይ ከሆነ ወደ 209 ጥያቄ ይለፉ | | | |
| 208 | | መልስ አዎ ከሆነ ጥ/ቁ 207 የደም ግፊት እንዳለብዎት ካወቁ በኋላ ጫት ቅመው ያውቃሉ? | | ሀ. አዎ  ለ. አይ | | |  | | | |
| 209 | | ቤተሰቦት ከሚጠቀሙት መጠን በመቀነስ ጨው ተጠቅመዋል? | | ሀ. አዎ  ለ. አይ | | |  | | | |
| 210 | | በምግብዎ ውስጥ ስብ/ጮማ ያላባባቸው ምግቦች ይቀንሳሉ?((ስጋ፣እንቁላለ፤ቂበ፤አይብ፤አሳ) | | ሀ. አዎ  ለ. አይ | | |  | | | |
| 211 | | አካላዊ እንቅስቃሴ አድርገው ያውቃሉ? | | ሀ. አዎ  ለ. አይ | | | መልስ አይ ከሆነ ወደ 301 ጥያቄ ይለፉ | | | |
| 212 | | መልስ አዎ ከሆነ ጥ/ቁ 212 የሚሰሩት አካላዊ እንቅስቃሴ ምን አይነት ነው( ከአንድ በላይ መልስመስጠት ይቻላል) | | ሀ. መጓዝ  ለ. በእግር መሮጥ  ሐ. ሳይክል መንዳት  መ. የቤት ውስጥ ስራዎች  ሠ.ሌሎች ካሉ ይገለጽ_______ | | |  | | | |
| 213 | | በሳምንት ለምን ያህል ቀናት አካላዊ እንቅስቃሴ ያደርጋሉ | | _____________ | | |  | | | |
| 214 | | በቀን ለምን ያህል ሰዓት አካላዊ እንቅስቃሴ ያደርጋሉ | | በሰዓት/በደቂቃ ________ | | |  | | | |
| 215 | | በየስንት ቀኑ ክትትል ያደርጋሉ? | | ሀ. በየወሩ  ለ. በየ2 ወሩ  ሐ.በየ3 ወሩ  መ. ሌላ ካለ ይገለጽ_______ | | |  | | | |
| 216 | | ቀጠሮ አሳልፈው ያውቃሉ?( ጤና ባላሙያ ቃጣሮ ኢንዳሳጡት) | | ሀ. አዎ  ለ. አይ | | |  | | | |
| **ክፍል 2.1። የሞሪስኪ መድኃኒት አዴራንስ መስፈርት** | | | | | | | | | | |
| ጥያቄ | | | | | | | አዎ | | አይ | |
| 301 | | አንዳንዴ መድሃኒት እረስተው ያውቃሉ? | | | | |  | |  | |
| 302 | | አንዳንድ ጊዜ ሰዎች መድኃኒታቸውን በመርሳት ሳይሆን በተላያየ ምክንያት ሊያቋርጡ ይችላሉ? በባለፉት ሁለት ሳምንታት ውስጥ እርስዎ መድኃኒት ያልወሰዱበት ቀን ያስታውሳሉ? | | | | |  | |  | |
| 303 | | በሚወስዱበት ጊዜ የባሰ ስሜት ስላደረብዎት ለሐኪምዎ ሳይናገሩ መድሃኒትዎን ቀንሰው ወይም አቋርጠው ያውቃሉ? | | | | |  | |  | |
| 304 | | ሲጓዙ ወይም ከቤት ሲወጡ አንዳንድ ጊዜ መድሃኒትዎን ይዘው መምጣት ይረሳሉ? | | | | |  | |  | |
| 305 | | ትናንት ሁሉንም መድሃኒትዎን ወስደዋል? | | | | |  | |  | |
| 306 | | ምልክቶችዎ በቁጥጥር ስር ያሉ እንደሆኑ ሲሰማዎት አንዳንድ ጊዜ መድሃኒትዎን መውሰድ ያቆማሉ? | | | | |  | |  | |
| 307 | | በየቀኑ መድሃኒት መውሰድ ለአንዳንድ ሰዎች ላይስማማ ይችላል ፡፡  የህክምና ዕቅድዎን ስለመፈጸም የመረበሽ ስሜት አጋጥሞዎት ያውቃል? | | | | |  | |  | |
| 308 | | ሁሉንም መድኃኒት አስታውሰው ለመውሰድ ምን ያህል ተቸግረው ያውቃሉ?( እባክዎን ትክክለኛውን መልስ ክብ ያድርጉ)  ሀ. በጭራሽ ለ. ከስንት አንዴ ሐ. አንዳንድ ጊዜ መ. ብዙውን ጊዜ ሠ. ሁልጊዜ | | | | |  | |  | |
| **ክፍል 3 ። የሕክምና መረጃ** | | | | | | | | | | |
| 401 | | የደም ግፊት እንዳለብዎ ካወቁ ምን ያህል ጊዜ ሆነዎት? | | | | | ______________________ | | | |
| 402 | | በቤተሰብ ውስጥ ስትሮክ/የነርቭ ህመም/ ያለበት ሰው አለ? | | | | | ሀ. አዎ  ለ. አይ | | | |
| **Data abstraction format from medical record** | | | | | | | | | | |
| 403 | | | | Type of stroke | | 1. Ischemic stroke 2. Hemorrhagic stroke | | | | |
| 404. | | | | Methods performed to diagnose stroke | | 1. CT-scan 2. MRI 3. Clinical | | | | |
| 405 | | | | Blood Pressure during first attack of stroke for cases or during data collection for controls | | 1. __________mmHg (during first attack of stroke or during the data collection for controls | | | | |
| 406 | | | | Blood Pressure before attacked of stroke for cases or before data collection for controls | | 1. _______mmHg (before attacked of stroke(cases) or before data collection for controls | | | | |
| 407 | | | | Laboratory results | | 1. Total cholesterol___________ 2. High density lipoprotein_____ 3. Low density lipoprotein_____ 4. Triglycerides_______________ | | | | |
| 408 | | | | Diabetic Mellitus | | 1. No 2. Yes | | | | |

**If you have any question you are welcome**

Thank you for your participation
