# Supplementary material for: Determinants of stroke among adult hypertensive patients on follow up in Addis Ababa public hospitals, Ethiopia: A case control study
Source: PLoS One. 2024 Sep 3;19(9):e0286845. doi: 10.1371/journal.pone.0286845 (PMC11371249; doi:10.1371/journal.pone.0286845)
Supplement: S3 File — (DOCX) [file pone.0286845.s003.docx]

**Unkaa 1ffaa: Afaan Oromo version questionnaires**

**Miiltoo A: Barreffama odeeffannoo hirmaattota filatamanii fi guca walii galtee fedhii irratti hundaa’e.**

**Akkam bultaan/oltan**

Ani maqaan koo _______________kanan jedhamu odeeffannoo qorannoo barataa Fayyisaa Tashooma barumsa digirii lammaffaa(maastersii) Yuunibersitii Ambootti muummee Meedisinii fi saanyinsii fayyaatti geggeesu kanan funaanudha. Dhaabbanni fayyaa keessan funaansa raga kanaatiif waan filatameef odeeffannoo barbaachisu akka naaf kennitan kabajaanan isin gaafaadha.

**Mata duree Qorannoo**

Sabaabota dhukkubsaattonni dhiibba dhiigaatin hospitaalota mootumma Finfinne keessatti yaalama jiran rakkoo dhiignii sammu keessatti akka dhangala’uuf(stroke) isaan mudatu taasisu adda baasu.

**Kaayyoo Qorannoo**

Hospitaalota Finfinnetti dhukkubsaattonni dhiibba dhiigattif deddebi’aani yaalama jiran rakkoo akka dhiignii sammutti dhangala’uu taasisu adda baasuf.

**Adeemsaa fi yeroo qorannichaa**

Qor-gaaffii fayyadamuun fayyadamtoota dhaabbillee fayyaa haasoofsisuun odeeffannoo gahaa funaannachuuf hedduu waan gargaaruuf. Gaaffillee kana deebisuuf yeroo keessan daqiiqaa 30 qofa fudhata.

**Miidhaa (Rakkoo) fi faayidaa qorannicha**

Rakkoon qorannoo kanarratti hirmaachuudhaan dhufu hin jiru, garuu yeroo keessan daqiiqaa muraasa qofa fudhata. Hirmaanna qorannoo kanaf waanti kaffalamu hin jiru. Garuu bu’aan qorannoo kanarraa argamu odeeffannoo barbaachisaa kan wiixinee fayyaaf oolu argamsiisa.

**Amantummaa**

Odeeffannoon ati asirratti nuuf kennitu kan itti amantu ta’uu qaba. Deebi ati kennituus eenyu akka kenne namni tokkolee beekuu hin danda’u. Lakkoofsi dhoksaa waan kennamuuf. Bu’aan isaatiis kan hawaasa walii gala fayyaduu dha.

**Mirga**

Hirmaataan fedhii isaatiin hirmaachu qaba. Dirqisiifamuu hin danda’u. yoo hirmaatees dhiisee bahuu fi deebisaa kennuu dhabuufiis mirga guutuu qaba. kan fedhe qofa deebisuufis mirga qaba.

**Wal qunnamsiiftuu/Teessoo/**

Yoo yaada, gaaffii fi kkf qabaattan teessoo kanaan quunnamuu dandeessu.

Maqaa Qorataa: Fayyisaa Tashooma

Lakkoofsa Moobaayilii: +251-9-21-40-63-87

E-mail- [fteshome3@gmail.com](mailto:fteshome3@gmail.com)

**Unka 2ffaa. Fedhii Hirmaataa Mirkanneessu**

Ani waa’ee qabiyyee hirmaataa sirritti dubbiisee/naaf dubbifamee jira. Faayidaa qo’annoo kanaa sirritti hubadheen jira. Akkaataa, faayidaafi miidhaa odeefannoo kennuu, mirga
hirmaataa fi teessoon ittin walqunnamee gaaffiin qabu gaafadhu,yoon fedhes qo’annoo kana
yeroon fedhetti dhiisee ba’uu akkan danda’u sirritti hubadheen jira.

Hirmaachuuf fedhii qabdaa? Eeyyee Lakkii

Mallattoo hirmaataa _____________________

Mallattoo nama odeeffannoo funaanee: ___________ Guyyaa___________

Mallattoo nama to’atuu: ______________________ Guyyaa___________

Lakkoofsa iccitii gaafi _______________________________

Maqaa Hospitaala ____________________

Lakkoofsa galmee dhukkubsata______________

Galatoomaa!

**Appendix E. Afaan Oromo Version Questionnaire**

| **Ajaja 1ffaa: Gaaffillee waa’ee haala jiruuf jireenya / hawwaasummaa dhukubsaata qorannoo irratti hirmaataniin walqabate. Hirmattotaa qorannoo irratti hirmaatanif gaaffi gochuun deebii lakkofsaa filannoo isaanii itti mari yookin bakka duwwa guuti.** | | | | | | | | | | | |  |
| --- | --- | --- | --- | --- | --- | --- | --- | --- | --- | --- | --- | --- |
| T.lakk | | | | Gaffile | | Deebii filannoo hirmaata | | | | Gara itti aanutti darbi | |  |
| 101. | | | | saala | | 1. Dhiira 2. Dhalaa | | | |  | |  |
| 102 | | | | Umuriin keessan meeqa? | | _____ waggadhan | | | |  | |  |
| 103 | | | | Amantaan keessan maali? | | 1. Ortodoksii 2. Proteestaantii 3. Musliima 4. Kaatolikii 5. Waaqeffataa 6. Kan biro (adda baasi)_____ | | | |  | |  |
| 104 | | | | Sadarkaan barnootaa keessan hangami? | | 1. Barumsaa ammayya kan hin qabne 2. Sadarkaa jalqabaa / 1-8/ 3. Sadarkaa 2 ^ffa^ (9-12) 4. Kooleejii fi isaa ol | | | |  | |  |
| 105 | | | | Hojiin keessan ammaa maali? | | \| 1. Qotte bulaa 2. Hojjetaa guyyaa 3. Hojii mootummaa 4. Hojji dhunfa 5. Dhaabbata mootummaan alaa (NGO) 6. Barattuu/taa 7. Haadha warraa 8. Kan biro (adda baasii)________ \| \| --- \| | | | |  | |  |
| 106 | | | | Haala gaa’elaa? | | 1. kan hin heerumne 2. Kan heerumte abba warraa waliin jirtu 3. Kan hiikte/ adda baate 4. Kan abbaan warraa jalaa du’e | | | |  | |  |
| 107 | | | | Iddoon jireenyaa keessan eessa? | | 1. Magaalaa 2. Baadiyyaa | | | |  | |  |
| **Ajaja 2ffa: Gaffilee Sababa amalaan waliqabatan** | | | | | | | | | | | |  |
| 201. | | Tamboo xuuxxee beektaa? | | | | | 1. Eeyyee 2. Lakkii | | | Deebiin lakkii yoo ta’e Gara G,203ti darbi | |  |
| 202. | | Deebiin keessan G 201 eeyeen yoo ta’ee, erga dhibba dhigaatiin qabamun kee baraame tamboo xuuxxee beekta? | | | | | 1. Eeyyee 2. Lakkii | | | Deebiin lakkii yoo ta’e Gara G,203ti darbi | |  |
| 203 | | Alkooli dhugdee beektaa? | | | | | 1. Eeyyee 2. Lakkii | | | Deebiin lakkii yoo ta’e Gara G,207ti darbi | |  |
| 204 | | Deebiin keessan G 203 eeyeen yoo ta’ee, erga dhibba dhigaatiin qabamun kee baraame alkooli dhugdee beekta? | | | | | 1. Eeyyee 2. Lakkii | | | Deebiin lakkii yoo ta’e Gara G,207ti darbi | |  |
| 205 | | Deebiin keessan G 204 eeyeen yoo ta’ee Gosa alkooli kam dhugda?(deebi heddu kennun nii danda’aama, deebi kenname hundatti maari) | | | | | 1. Biiraa 2. Wayinii 3. “Farsoo” 4. “daadhii/xajjii” 5. “Araqee/katikaala” 6. Kan biroo_______ | | |  | |  |
| 206 | | Guyyaatti giddugaleessan alkooli meeqa dhugda? (xarmussi/qaruradhan, burcuqoodhan, kubbayya, “malaka” ) | | | | | ________________  __________________  __________________ | | |  | |  |
| 207 | | Caati qamaate beektaa? | | | | | 1. Eeyyee 2. Lakkii | | | Deebiin lakkii yoo ta’e Gara G,209ti darbi | |  |
| 208 | | Deebiin keessan G 207 eeyeen yoo ta’ee Ergaa dhiibbaa dhigaaa qabaachun kee baraame caati qamaate beektaa? | | | | | 1. Eeyyee 2. Lakkii | | |  | |  |
| 209 | | Soogida hangaa maatin kee sooratanirra hiri’stee nii sooratta? | | | | | 1. Eeyyee 2. Lakkii | | |  | |  |
| 210 | | Nyaata cooma fi bu’aa looni qaban nii nyaatta?(kan akka foon, kille, dhaadha, qurxummi, kireemi, fi kkf) | | | | | 1. Eeyyee 2. Lakkii | | |  | |  |
| 211 | | Sochii qaama nii tasistaa? | | | | | 1. Eeyyee 2. Lakkii | | | Deebiin lakkii yoo ta’e Gara G,215ti darbi | |  |
| 212 | | Deebin G, 211tif Eyyen yoo ta’ee, sochii qaama gosaa kami tasistaa? ?(deebi heddu kennun nii danda’aama, deebi kenname hundatti maari) | | | | | 1. Deemsaa milaa 2. Suksukii 3. Sayikilii oofu 4. Hojji mana keessa 5. Kan biroo­­­­­­­­______ | | |  | |  |
| 213 | | Torbanitti guyyota meeqa sochii qaama tasistaa? | | | | | _____________ | | |  | |  |
| 214 | | Guyyatti daqiqaa/sa’aaati meqaf sochii qaama taasista? | | | | | Daqiqaa /sa’aatidhan___________ | | |  | |  |
| 215 | | Hordooffin yaalaa keessaan yeroo meeqan? | | | | | 1. Ji’aan 2. Ji’aa 2’n 3. Ji’aa 3’fi issa ol | | |  | |  |
| 216 | | Hordooffi yaalaa keessan addan kuttanni beektuu?(akkaataa ogeessi fayyaa isin beellameen) | | | | | 1. Eeyyee 2. Lakkii | | |  | |  |
| **Ajajaa 2.1: Gaffilee Morisky Medication adherence standard checklistin wal-qabatan** | | | | | | | | | | | |  |
| Gaffilee | | | | | | | | | | eyyee | lakki |  |
| 301 | | | | | Yeroo tokko tooko qorichaa kee fudhachu nii irraanfattaa? | | | | |  |  |  |
| 302 | | | | | Namoonni yeroo tokko tokkko qorichaa isaani osso hin irranfaatin sababaa wayyitif fudhaachu nii dhisu. Torbaanota laman darbaan yaadadhuti guyyaan atii qorichaa hin fudhaatin nii jiraa? | | | | |  |  |  |
| 303 | | | | | Qorichaa keessaan osoo fudhachaa jirtaanu sabaaba mirrii badaan isinitti dhagahamef ossoo ogeessa fayyaatti hin himiin qorichaa hiri’iistani fudhaatani yookin addaa kuttaani beektu? | | | | |  |  |  |
| 304 | | | | | Yeroo tokko tokkoo yoo manaa baatan yookin karaa deemtan qorichaa osoo hin qabatin irranfattani deemtanii beektu? | | | | |  |  |  |
| 305 | | | | | Kaalleessa qorichaa keessaan hundumaa liqimsitani/fudhaatani? | | | | |  |  |  |
| 306 | | | | | Mallatton dhukubaa keeti to’aanna jalaa oolu yoo sitti dhagahamu, yeroo tokko tokko qorichaa liqimsuu nii dhistaa? | | | | |  |  |  |
| 307 | | | | | Guyyaa guyyaadhan namoota tokko tokkotti qorichaa fudhaachun itti tolu dhiisu danda’aa. Karoora yaala keessaani galmaan ga’uuf mirrii jeeqamu isiin muudatee beekaa? | | | | |  |  |  |
| 308 | | | | | Qorichaa keessaan hundumaa yaadattani liqimsudhaf/ fudhaachudhaf ammam rakkattani beektu? ( **Deebi sirrii ta’eetti mari)** 4. Goonkuma 3. Yeroo meeqatti al tokko. 2. Darbe darbee 1. Yeroo hedduu 0. Yeroo hundaa | | | | |  |  |  |
| **Ajaaja 3ffa. Odeeffanno yaalaa** | | | | | | | | | | | | |
| 401 | | Erga dhiibbaa dhigatiin qabamu keesssan bartaani/baraame ammam turee?? | | | | | | | | ___________ | | |
| 402 | | Maati keessan keessa kan dhukuba sammuu keessaatti dhangala’uu dhiiga(stroke) qabu jira? | | | | | | | | 1. Yes 2. No | | |
| **Data abstraction format from medical record** | | | | | | | | | | | | |
| 403 | | | | Type of stroke | | | | | 1. Ischemic stroke 2. Hemorrhagic stroke | | | |
| 404. | | | | Methods performed to diagnose stroke | | | | | 1. CT-scan 2. MRI 3. Clinical | | | |
| 405 | | | | Blood pressure during first attack of stroke for cases or during data collection for controls | | | | | 1. __________mmHg (during first attack of stroke or during data collection for controls. | | | |
| 406 | | | | Blood pressure before attacked of stroke for cases or before data collection for controls | | | | | 1. ________mmHg (before attacked of stroke(cases) or before data collection for (controls) patients | | | |
| 408 | | | | Laboratory results | | | | | 1. Total cholesterol____________ 2. High density lipoprotein______ 3. Low density lipoprotein______ 4. Triglycerides____________ | | | |
| 409 | | | | Diabetic Mellitus | | | | | 1. No 2. Yes | | | |
